# How will COVID-19 transform global health post-pandemic? Defining research and investment opportunities and priorities

**DOI:** 10.1371/journal.pmed.1003564

**Published:** 2021-03-11

**Authors:** Michael Reid, Quarraisha Abdool-Karim, Elvin Geng, Eric Goosby

**Affiliations:** 1 Institute for Global Health Sciences, University of California, San Francisco, California, United States of America; 2 Centre for the AIDS Programme of Research in South Africa (CAPRISA), Durban, Kwa-Zulu Natal, South Africa; 3 Department of Epidemiology, Columbia University, New York, New York, United States of America; 4 Department of Medicine, and the Center for Dissemination and Implementation, Institute for Public Health, Washington University, St. Louis, Missouri, United States of America

## Abstract

Michael Reid and co-authors introduce a Collection on the global health in the post-COVID-19 era.

COVID-19 represents the greatest threat to global public health and economies in the 21^st^ century. To date, more than 100 million people have been infected and over 2.1 million died; likely the tip of the iceberg of this devastating pandemic [1]. The impact on other pandemics and health challenges is masked by the continued dominance of COVID-19 on our lives. The economic and social impact is formidable and growing. In the United States (US) alone, the pandemic’s total cost is estimated at more than US$16 trillion, or approximately 90% of the country’s annual gross domestic product [2]. No country or region has been spared; the crisis has laid bare stark weaknesses in almost every health care system. Within and between countries we have witnessed how already vulnerable and marginalized populations bear a disproportionate burden of infection, with reversal of the development gains made to date [3].

The pandemic has also highlighted our interdependence. To achieve a new normal requires a coordinated, coherent and cohesive response that is effective globally for sustainability. For decades to come, we will undoubtedly continue critical reflection on why some countries, including less-resourced countries, have been able to respond more effectively to COVID-19, while some well-resourced countries have struggled. For now, a high priority in ending the pandemic, as well as accelerating the post-COVID-19 recovery, must be determining what an effective global response to COVID-19 entails. The papers forming this PLOS Special Collection, *Post pandemic*: *How will COVID-19 transform global health*? were conceived as a road-map to catalyse a global health research and policy agenda necessary to end the pandemic, shape the post-COVID-19 global health recovery and lay the foundations for a more resilient global landscape to achieve the goals of sustainable development. While by no means comprehensive, we highlight several critical themes: investments in better pandemic preparedness; more invigorated efforts to address structural and systemic inequities within the sustainable development framework and universal health care; enhancing resilience of our health care delivery systems; and achieving greater accountability for our actions as a threat to one poses a threat to all.

As papers in this Collection outline, COVID-19 has underscored the need for sustained investment in global health research. The speed and scale of vaccine development efforts, the proliferation of diagnostic tools and the development of several proven and promising therapeutic agents, are all evidence of where global science has triumphed and reflect decades of investments in biomedical research that could be rapidly brought to bear in the face of this new threat. Nonetheless, SARS-CoV-2 was a virus we should have been ready for; after all, we have been planning and preparing since the 1918 influenza pandemic. We had already encountered the SARS and MERS pandemics: these coronaviruses were already on the WHO Blueprint program priority list, large-scale studies had already identified a broad range of SARS-like coronaviruses in the potential reservoirs [4], and tools for structural analysis and prediction of host-range were ready [5]. So why were we not better prepared for this pandemic and able to contain the global transmission or correctly predict host-range? At the global level, preparations were inadequate and fragmented. Moreover, coordinated, transparent and inclusive global research is critical to inform future pandemic preparedness. Translating and expanding our existing knowledge on reservoirs, viruses, and drivers for disease emergence into a program for early warning and predictive outbreak risk is likely to be challenging, but absolutely imperative to mitigate against future pandemics, and will demand coordinated, well-funded global health and resourced research efforts.

Second, COVID-19 has amplified long-standing systemic and structural global health inequities [6], including in poverty, access to health care, race, ethnicity, gender and social incohesion. Manuscripts in this Collection seek to highlight how addressing these inequities must be both a local and a global priority. While the consequences of deaths from COVID-19 in East Los Angeles are consequences for California, they are also consequences for the rest of the world, especially given the proclivity of this virus for mutation and transmission. Renewed attention must be focused on how global health efforts that address local health inequities are integral to addressing global disparities. Even before the coronavirus pandemic, social, economic and health inequities had become a prevailing global narrative, with increasing scholarly attention focused on how global health needed to be de-colonized to redress power imbalances that perpetuate these inequities within and between nations [7,8]. Citizens globally are more visibly expressing anger and intolerance of enduring health, social, and economic injustices. Structural inequities reproduced within the global health system itself highlight the lack of critical engagement with the political and social determinants of health disparities [9]. While the global response to COVID-19 has largely reinforced these injustices, post-COVID-19 there is an opportunity to transform global health through an agenda of repoliticising and rehistoricizing health, building on the renewed and critical awareness brought to attention by the current pandemic [8].

Third, COVID-19 has served as a ‘stress test’ for health systems the world over. Some resilient health systems have responded rapidly and effectively to the pandemic, while many others have not. The salutary performance of East Asian countries in responding to the current pandemic, explained in part by imprinting and learning from past outbreaks, may offer a model for how health security must be prioritized in the post-COVID-19 era. By contrast, the dysfunctional performance of a few high-income nations illustrates the perils of under-investing in public health systems to cope with health crises of this scale. Equitable access to vaccines in the recovery process will also be an important stress test of global collaboration in the face of rising vaccine nationalism [10]. Developing safe and effective vaccines alone will not be enough to end the pandemic, unless those vaccines can be delivered globally at a price affordable to all governments and allocated in a way that maximizes public health impact and achieves equity. This Collection will explore the role of the COVID-19 Vaccine Global Access Facility (COVAX) initiative in encouraging high-income nations to participate to avoid a ‘tragedy of the commons’ [11] by expanding global vaccine supply and delivery, while also generating other positive outcomes [12].

Achieving rapid pandemic control is possible but is predicated on decisive leadership and collaboration for COVID-19 responses—at global, national and local levels—and a commitment to leave no one behind. Global health diplomacy has a critical role to play in catalysing governments and non-state actors to enact effective, innovative and just policy solutions. An ‘every country for itself’ approach clearly does not hold up in this interdependent world, and we need reforms. In particular, the International Health Regulations, which govern all countries to have core health system capacities to detect future pandemics, need to be revitalized. More effective rules of the road are also needed to foster cooperation among countries seeking to manage future outbreaks and as a global trigger for the United Nations and other international organizations to take appropriate actions. A well-funded WHO is also essential to effective global health governance and offers a model for global collaboration. The WHO has a critical role to play in supporting all countries to prioritize universal health systems, not only because it will vastly improve health and be an important bulwark against future pandemics, but also to reap marked economic dividends.

Although we have much to learn about SARS-CoV-2, the epidemic and its consequences, the virus has made one thing clear: for any crisis that threatens the globe, the problems of any of us are the problems of all of us. Global post-pandemic recovery must therefore be coordinated and multi-dimensional. Governance systems that are inclusive, accountable and guided by approaches that prioritize transparent, multisectoral decision-making processes are urgently needed to respond effectively. Only a holistic response based on cross-sectoral collaboration at all levels of society can build the necessary resilience to respond to the immediate and long-term effects of COVID-19. The COVID-19 pandemic reminds us that no country acting alone can respond effectively to health threats in a globalized world [13]. The crisis has also created a unique opportunity to re-imagine and transform global health so that future pandemics are not nearly as devastating as this one, and that health gains made to date are sustained and strengthened rather than reversed.
